# Sodium bis­(ethyl­enedi­amine)­copper(II) tetra­cyanido­cuprate(I)

**DOI:** 10.1107/S1600536813012075

**Published:** 2013-05-11

**Authors:** Peter W. R. Corfield, Robert K. Dobbs, Brian Bell

**Affiliations:** a Department of Chemistry, Fordham University, 441 East Fordham Road, Bronx, NY 10458, USA; b The King’s College, Briarcliff Manor, NY 10510, USA

## Abstract

The title compound, Na[Cu(en)_2_][Cu(CN)_4_], where en represents ethyl­enedi­amine, NH_2_CH_2_CH_2_NH_2_, crystallizes as a salt with two distinct cations, Na^+^ and [Cu^II^en_2_]^2+^, and discrete [Cu^I^(CN)_4_]^3−^ anions. The anion geometry is tetra­hedral, with angles at the copper atom ranging from 105.0 (1) to 115.4 (1)°. The Cu—C distances are in the range 1.976 (3) to 1.993 (3) Å. The divalent copper atom is coordinated by four N atoms of the two bidentate en ligands in a slightly distorted square-planar geometry. In the crystal, each sodium ion inter­acts with cyanide N atoms of four different anions, with Na—N distances lying in the narrow range of 2.344 (3) to 2.367 (3) Å, and an approximately tetra­hedral arrangement around the sodium ions. The inter­acting sodium ions and [Cu^I^(CN)_4_]^3−^ anions form a three-dimensional network with channels which contain the [Cu(en)_2_]^2+^ cations. One of the chelate rings in the cation shows partial disorder between two different conformations and the C atoms were refined with occupancies in the ratio 0.817 (15):0.183 (15).

## Related literature

The work presented here continues studies on mixed-valence copper cyanide complexes, see: Corfield *et al.* (2012 ▶). Studies by others on similar complexes include Colacio *et al.* (2002 ▶) and Kim *et al.* (2005 ▶). For other results on the specific system Cu^I,II^—CN—en, see: Williams *et al.* (1972 ▶) and Weiss *et al.* (2006 ▶). We are aware of only one other detailed crystal structure describing the discrete [Cu(CN)_4_]^3−^ anion, that reported for K_3_Cu(CN)_4_ in Roof *et al.* (1968 ▶). For molar conductance, see: Angelici (1977 ▶).
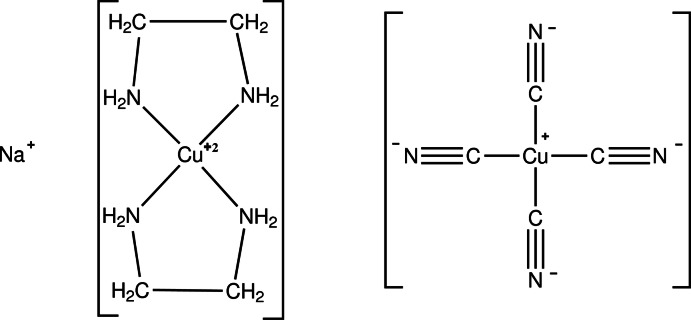


## Experimental

### 

#### Crystal data

Na[Cu(C_2_H_8_N_2_)_2_][Cu(CN)_4_]
*M* *_r_* = 374.36
Monoclinic, 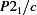

*a* = 8.842 (1) Å
*b* = 10.743 (1) Å
*c* = 15.268 (3) Å
β = 98.32 (1)°
*V* = 1435.0 (4) Å^3^
*Z* = 4
Cu *K*α radiation
μ = 3.94 mm^−1^
*T* = 295 K
0.33 × 0.27 × 0.16 mm

#### Data collection

Enraf–Nonius CAD-4 diffractometer
Absorption correction: integration Busing & Levy (1957 ▶) *T*_min_ = 0.361, *T*_max_ = 0.576
5288 measured reflections
2679 independent reflections
2614 reflections with *I* > 2σ(*I*)
*R*_int_ = 0.024
3 standard reflections every 120 min intensity decay: 11%

#### Refinement

*R*[*F* ^2^ > 2σ(*F* ^2^)] = 0.029
*wR*(*F* ^2^) = 0.087
*S* = 1.19
2679 reflections
181 parameters
H-atom parameters constrained
Δρ_max_ = 0.32 e Å^−3^
Δρ_min_ = −0.20 e Å^−3^

### 

Data collection: *CAD-4 Software* (Enraf–Nonius, 1994 ▶); cell refinement: *CAD-4 Software*; data reduction followed procedures in Corfield *et al.* (1973 ▶): data were averaged with a local version of *SORTAV* (Blessing, 1989 ▶); program(s) used to solve structure: locally modified program (Corfield, 1984 ▶); program(s) used to refine structure: *SHELXL97* (Sheldrick, 2008 ▶) and *XABS2* (Parkin *et al.*, 1995 ▶); molecular graphics: *ORTEPIII* (Burnett & Johnson, 1996 ▶); software used to prepare material for publication: *SHELXL97*.

## Supplementary Material

Crystal structure: contains datablock(s) global, I. DOI: 10.1107/S1600536813012075/lh5608sup1.cif

Structure factors: contains datablock(s) I. DOI: 10.1107/S1600536813012075/lh5608Isup2.hkl

Additional supplementary materials:  crystallographic information; 3D view; checkCIF report

## Figures and Tables

**Fig. 1.**
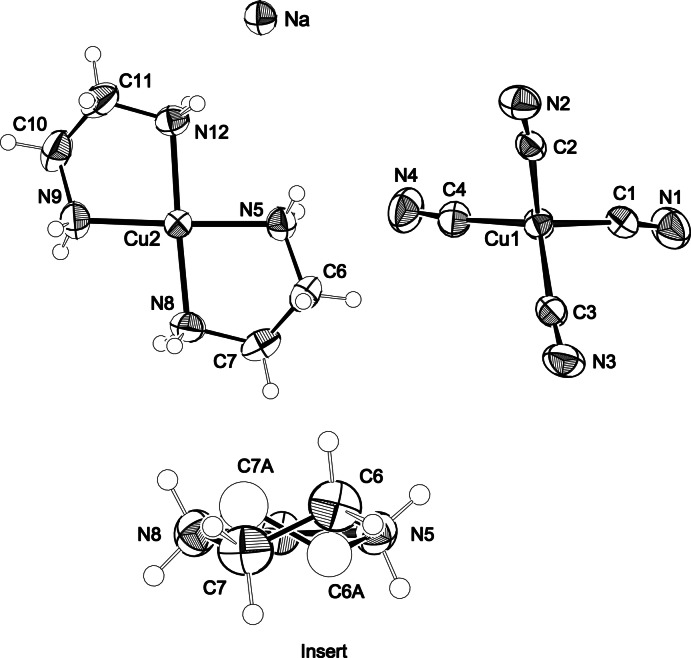
The cations and anions in Na[Cuen2][Cu(CN)4], with ellipsoids at the 50% level. Relative positions and orientations of these ions were altered to give a convenient figure. The insert shows the disordered chelate ring, looking into the N8—Cu2—N5 plane. The minor component was refined isotropically. Hydrogen atoms on C6A and C7A are deleted for clarity.

**Fig. 2.**
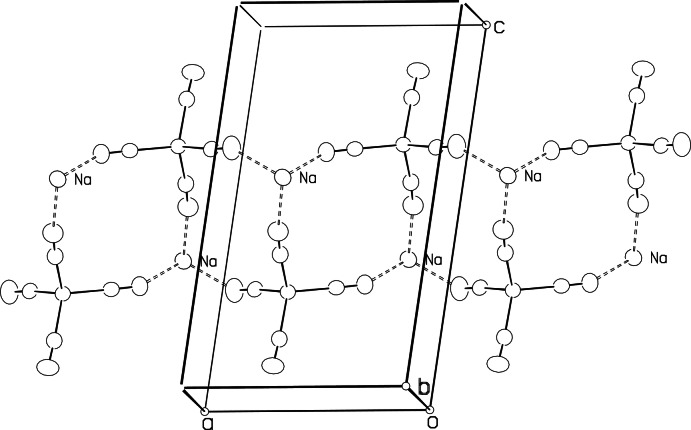
One of the ribbons formed by interactions between sodium ions and the [Cu(CN)4]3- anions. Ellipsoids are at the 50% level.

**Fig. 3.**
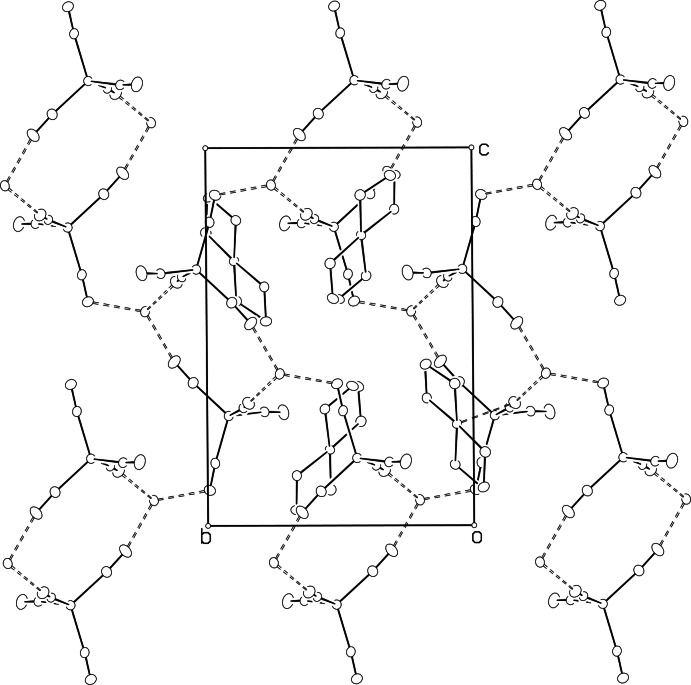
View of the channels along a formed by the ribbons formed by sodium ion to [Cu(CN)4]3- interactions. Each channel contains two planar [Cuen2]2+ cations per unit cell.

## supplementary crystallographic information

### Comment

There has been widespread interest in structures of cyanide-bridged copper compounds, which can show self-assembly into polymeric structures. See, for example, Colacio *et al.* (2002) and Kim *et al.* (2005). The structure determination of the title compound was undertaken as part of a series of synthetic and structural studies of mixed-valence copper cyanide complexes containing chelating amine bases (Corfield *et al.*, 2012). The coordinated amines stabilize the divalent copper atoms against reduction by the cyanide groups. The title compound was crystallized from systems with the base ethylenediamine. The structures of two other compounds from this system have been described (Williams *et al.*, 1972; Weiss *et al.*, 2006).

The crystal structure of the title compound consists of sodium and bisethylenediamine copper^II^ cations, Na^+^ and [Cuen_2_]^2+^, crystallizing with discrete tetracyanidocopper^I^ anions, [Cu(CN)_4_]^3-^, (Fig. 1). The divalent copper atom, Cu2, shows square planar coordination with the four N atoms of the two bidentate ligands, with average Cu—N distances of 2.004 (2) Å. The Cu2–N5–C6–C7–N8 chelate ring has the λ conformation, with torsional angle of -50.3 (6)°. The disordered chelate ring Cu2–N5–C6A–C7A–N8 has the opposite δ conformation, as does the Cu2–N9–C10–C11–N12 chelate ring, with torsion angle +53.0 (3)°.

The monovalent copper atom, Cu1, shows tetrahedral coordination to the carbon atoms of four cyanide groups, with bond angles averaging 109.5°. The Cu—C distances lie within the narrow range of 1.976 (3)–1.993 (3) Å, similar to the Cu—C distances of 2.014 (10) Å and 1.993 (6) Å reported by Roof *et al.*, (1968). There is no ambiguity between cyanide carbon and nitrogen atoms. The C—Cu—C angles range from 101.8 (1)° to 115.4 (1)°.

There are significant interactions between the sodium ions, Na^+^, and the nitrogen atoms of CN groups from four different [Cu(CN)_4_]^3-^ anions, with an average Na—-N distance of 2.358 (2) Å. The arrangement of cyanide N atoms around the sodium ions is roughly tetrahedral, with angles ranging from 100.8 (1)° to 129.8 (1)°. The C—N—Na angles average 140.8°, with wide variation from 113.3 (2)° to 165.3 (2)°, unlike the linear arrangement that would be expected for covalently bridging cyanide groups.

Three of the four Na—N interactions link the Na^+^ cations and the [Cu(CN)_4_]^3-^ anions into ribbons extending along the direction of the *a* axis, (Fig. 2) while a fourth Na—N interaction links the ribbons into an open three-dimensional structure with channels along the *a* axis. The [Cuen_2_]^2+^ cations reside in these channels (Fig. 3). There appear to be significant interactions between the Cu atoms of the cation and cyanide N atoms from two neighboring [Cu(CN)_4_]^3-^ anions, with N1 and N2 atoms of the anions lying approximately in the axial positions for the square planar cationic copper atoms, at distances of 3.09 Å and 2.69 Å respectively. We have not described the latter interaction as a long covalent bond, because the putative Cu—N—C angle is 99.0°, far different from the linear angle expected for a bonded cyanide group. Further, the Cu atom is displaced very little from the N_4_ plane of the coordinated ethylenediamine ligands.

Three possible weak N—H···N hydrogen bonds link N8 and N9 atoms from the ethylenediamine ligands with neighboring cyanide N atoms, with N···N distances ranging from 3.30 to 3.53 Å, N···H distances from 2.64 to 2.67 Å, and angles at the hydrogen atoms ranging from 132° to 165°. Other intermolecular contacts appear normal, with the shortest H···H intermolecular distance found at 2.58 Å, and the shortest H···C distance at 2.41 Å, for H5B—C3(1 - *x*, -1/2 + *y*, 1/2 - *z*).

### Experimental

The compound was prepared by addition of ~13 ml of a solution containing 8.0 g (0.133 mol) of ethylenediamine to a solution containing 8.34 g (0.17 mol) sodium cyanide and 8.17 g (0.09 mol)copper(I) cyanide in 33 ml water. Crystals usually appear after several days. Often, the first compound filtered off is Cu_3_en_2_(CN)_4_H_2_O (Williams *et al.*, 1972), while later batches provide samples of the present compound, Na[Cuen_2_][Cu(CN)_4_].

Sodium was analyzed by atomic absorption spectroscopy, with a Perkin-Elmer 303 instrument. Found: 6.0 (2)%; calculated 6.15%. Total copper was analyzed iodometrically: found 33.8 (1)% from 13 measurements; calculated 34.0%. Cyanide was analyzed by titration with AgNO_3_: found 26.7(1.7)% from two measurements; calculated 27.8%. The infra-red spectrum was taken with a Perkin- Elmer 710B spectrophotometer. There is one CN stretching frequency, at 2095 cm^-1^. Conductance measurements with a classical conductance bridge and glass cell gave a molar conductance of 293 (2) ohm^-1^.cm^2^.mol^-1^, a reasonable value for a three ion electrolyte (Angelici, 1977).

### Refinement

Refinements with anisotropic temperature factors for Cu, N and C atoms and isotropic factors for the constrained hydrogen atom parameters converged smoothly. H atoms were placed in calculated positions with N—H = 0.90Å and C—H = 0.97Å. They were included in the refinment in a riding motion approximation with U_iso_(H) = 1.2U_eq_(C,N) or 1.5U_eq_(C) for disordered atoms. At this point, a difference Fourier synthesis showed peaks of 0.6 e/A^3^ that indicated partial occupancy of an alternative conformation δ for the ethylenediamine carbon atoms C6 and C7, which have a λ conformation. Further refinements allowed for disorder of this ethylenediamine group. The extra atoms were labeled C6A and C7A and were refined isotropically, with hydrogen atoms assigned in constrained positions. The nitrogen atoms N5 and N8 were assumed to be common to both conformations. The main conformation, involving C6 and C7, refined to an occupancy of 81.7 (15)%, while the alternative conformation with C6A and C7A has an occupancy of 18.3 (15)%. Inclusion of the disordered atoms in the refinement lowered R1 from 0.0339 to 0.0322.

A difference Fourier synthesis at this point showed several holes of depth -0.6 e/A^3^ within 1 A of the copper atoms. It was felt that these features might reflect small anisotropic errors in the diffracted intensities due to the size of the crystal relative to the size of the fine focus X-ray beam, as well as a slight crystal movement noted towards the end of the data collection. The anisotropy was modeled by using the program *XABS2*, (Parkin *et al.*, 1995). Twelve parameters were used to modify the observed structure factors. Subsequent refinements lowered R1 from 0.0322 to 0.0300 for all reflections, and reduced the maximum height or depth of features in the final difference Fourier synthesis to 0.32 e/A^3^ or less.

### Crystal data

**Table:**

Na[Cu(C_2_H_8_N_2_)_2_][Cu(CN)_4_]	*F*(000) = 756
*M**_r_* = 374.36	*D*_x_ = 1.733 Mg m^−^^3^*D*_m_ = 1.732 (3) Mg m^−^^3^*D*_m_ measured by flotation in 1-bromobutane/1,1,4,4-tetrabutane mixtures. Four independent determinations were made.
Monoclinic, *P*2_1_/*c*	Cu *K*α radiation, λ = 1.5418 Å
Hall symbol: -P 2ybc	Cell parameters from 25 reflections
*a* = 8.842 (1) Å	θ = 2.7–24.2°
*b* = 10.743 (1) Å	µ = 3.94 mm^−^^1^
*c* = 15.268 (3) Å	*T* = 295 K
β = 98.32 (1)°	Block, dark blue
*V* = 1435.0 (4) Å^3^	0.33 × 0.27 × 0.16 mm
*Z* = 4	

### Data collection

**Table:**

Enraf–Nonius CAD-4 diffractometer	2614 reflections with *I* > 2σ(*I*)
Radiation source: fine-focus sealed tube	*R*_int_ = 0.024
Graphite monochromator	θ_max_ = 70.2°, θ_min_ = 5.1°
θ/2θ scans	*h* = −10→10
Absorption correction: integration Busing & Levy (1957)	*k* = 0→11
*T*_min_ = 0.361, *T*_max_ = 0.576	*l* = 0→18
5288 measured reflections	3 standard reflections every 120 min
2679 independent reflections	intensity decay: 11%

### Refinement

**Table:**

Refinement on *F*^2^	Primary atom site location: heavy-atom method
Least-squares matrix: full	Secondary atom site location: difference Fourier map
*R*[*F*^2^ > 2σ(*F*^2^)] = 0.029	Hydrogen site location: inferred from neighbouring sites
*wR*(*F*^2^) = 0.087	H-atom parameters constrained
*S* = 1.19	*w* = 1/[σ^2^(*F*_o_^2^) + (0.*P*)^2^ + 1.160*P*] where *P* = (*F*_o_^2^ + 2*F*_c_^2^)/3
2679 reflections	(Δ/σ)_max_ < 0.001
181 parameters	Δρ_max_ = 0.32 e Å^−^^3^
0 restraints	Δρ_min_ = −0.20 e Å^−^^3^

### Special details

**Table:**

Experimental. Diffraction data were collected with Cu *K*α radiation. At a later stage, Mo *K*α radiation was used in measurements made to obtain improved unit-cell dimensions. Each of the 25 reflections used for cell measurement was determined in four different orientations using the CAD4 *SET4* command.
Geometry. All e.s.d.'s (except the e.s.d. in the dihedral angle between two l.s. planes) are estimated using the full covariance matrix. The cell e.s.d.'s are taken into account individually in the estimation of e.s.d.'s in distances, angles and torsion angles; correlations between e.s.d.'s in cell parameters are only used when they are defined by crystal symmetry.
Refinement. Refinement of *F*^2^ against ALL reflections. The weighted *R*-factor *wR* and goodness of fit *S* are based on *F*^2^, conventional *R*-factors *R* are based on *F*, with *F* set to zero for negative *F*^2^. The threshold expression of *F*^2^ > σ(*F*^2^) is used only for calculating *R*-factors(gt) *etc*. and is not relevant to the choice of reflections for refinement. *R*-factors based on *F*^2^ are statistically about twice as large as those based on *F*, and *R*- factors based on ALL data will be even larger.

### Fractional atomic coordinates and isotropic or equivalent isotropic displacement parameters (Å^2^)

**Table:**

	*x*	*y*	*z*	*U*_iso_*/*U*_eq_	Occ. (<1)
Cu1	0.71472 (4)	−0.04991 (3)	0.31263 (2)	0.03584 (13)	
Cu2	0.24744 (4)	0.07238 (3)	0.27612 (2)	0.03475 (13)	
Na	0.22615 (12)	−0.26385 (10)	0.40880 (7)	0.0409 (2)	
N1	0.9771 (3)	−0.2440 (3)	0.3300 (2)	0.0638 (8)	
C1	0.8783 (3)	−0.1771 (2)	0.32825 (17)	0.0371 (5)	
N2	0.3881 (3)	−0.1387 (3)	0.33609 (18)	0.0526 (6)	
C2	0.5099 (3)	−0.1134 (2)	0.32682 (16)	0.0353 (5)	
N3	0.7238 (4)	0.0236 (3)	0.11626 (17)	0.0588 (7)	
C3	0.7155 (3)	0.0020 (2)	0.18738 (18)	0.0384 (6)	
N4	0.7729 (3)	0.1558 (3)	0.4577 (2)	0.0632 (7)	
C4	0.7567 (3)	0.0847 (3)	0.4020 (2)	0.0435 (6)	
N5	0.3599 (3)	0.0853 (2)	0.17195 (15)	0.0401 (5)	
H5A	0.3405	0.1593	0.1451	0.048*	
H5B	0.4612	0.0797	0.1899	0.048*	
C6	0.3095 (6)	−0.0168 (5)	0.1094 (3)	0.0489 (14)	0.817 (15)
H6A	0.3605	−0.0937	0.1296	0.073*	0.817 (15)
H6B	0.3348	0.0027	0.0512	0.073*	0.817 (15)
C7	0.1402 (6)	−0.0307 (5)	0.1052 (3)	0.0508 (13)	0.817 (15)
H7A	0.0884	0.0403	0.0752	0.076*	0.817 (15)
H7B	0.1057	−0.1054	0.0726	0.076*	0.817 (15)
C6A	0.257 (3)	0.034 (2)	0.0970 (12)	0.047 (5)*	0.183 (15)
H6A1	0.3137	0.0120	0.0496	0.070*	0.183 (15)
H6A2	0.1804	0.0958	0.0747	0.070*	0.183 (15)
C7A	0.181 (3)	−0.077 (3)	0.1271 (17)	0.055 (6)*	0.183 (15)
H7A1	0.1095	−0.1110	0.0791	0.083*	0.183 (15)
H7A2	0.2567	−0.1401	0.1472	0.083*	0.183 (15)
N8	0.1045 (3)	−0.0388 (2)	0.19709 (16)	0.0446 (5)	
H8A	0.1150	−0.1179	0.2164	0.053*	
H8B	0.0072	−0.0151	0.1984	0.053*	
N9	0.1297 (3)	0.0750 (2)	0.37895 (17)	0.0469 (6)	
H9A	0.0287	0.0782	0.3595	0.056*	
H9B	0.1493	0.0057	0.4118	0.056*	
C10	0.1775 (4)	0.1856 (3)	0.4322 (2)	0.0555 (8)	
H10A	0.1477	0.1779	0.4906	0.083*	
H10B	0.1295	0.2595	0.4041	0.083*	
C11	0.3480 (4)	0.1955 (3)	0.43916 (19)	0.0552 (8)	
H11A	0.3830	0.2729	0.4678	0.083*	
H11B	0.3963	0.1270	0.4740	0.083*	
N12	0.3880 (3)	0.1914 (2)	0.34902 (14)	0.0396 (5)	
H12A	0.4855	0.1663	0.3508	0.048*	
H12B	0.3789	0.2677	0.3246	0.048*	

### Atomic displacement parameters (Å^2^)

**Table:**

	*U*^11^	*U*^22^	*U*^33^	*U*^12^	*U*^13^	*U*^23^
Cu1	0.0382 (2)	0.0338 (2)	0.0355 (2)	−0.00035 (15)	0.00530 (16)	−0.00042 (14)
Cu2	0.0366 (2)	0.0353 (2)	0.0331 (2)	−0.00367 (14)	0.00756 (15)	−0.00332 (14)
Na	0.0462 (6)	0.0331 (5)	0.0447 (6)	−0.0020 (4)	0.0107 (4)	−0.0033 (4)
N1	0.0515 (15)	0.0500 (16)	0.091 (2)	0.0087 (13)	0.0137 (14)	0.0131 (15)
C1	0.0375 (13)	0.0344 (14)	0.0385 (13)	−0.0029 (11)	0.0020 (10)	0.0027 (10)
N2	0.0444 (14)	0.0560 (16)	0.0578 (15)	−0.0038 (12)	0.0089 (11)	0.0106 (12)
C2	0.0429 (14)	0.0289 (13)	0.0335 (12)	0.0004 (10)	0.0038 (10)	0.0037 (10)
N3	0.084 (2)	0.0511 (16)	0.0416 (14)	−0.0138 (14)	0.0114 (13)	0.0056 (12)
C3	0.0405 (13)	0.0319 (13)	0.0430 (14)	−0.0007 (11)	0.0062 (11)	0.0026 (11)
N4	0.0684 (18)	0.0588 (18)	0.0638 (17)	−0.0049 (14)	0.0139 (14)	−0.0237 (15)
C4	0.0415 (14)	0.0421 (15)	0.0470 (15)	0.0001 (12)	0.0070 (12)	−0.0039 (12)
N5	0.0438 (12)	0.0387 (12)	0.0395 (11)	0.0005 (9)	0.0112 (9)	0.0028 (9)
C6	0.058 (3)	0.055 (3)	0.0351 (18)	0.003 (2)	0.0101 (16)	−0.0093 (17)
C7	0.059 (2)	0.051 (3)	0.039 (2)	0.000 (2)	−0.0056 (17)	−0.0091 (18)
N8	0.0418 (12)	0.0447 (13)	0.0452 (13)	−0.0075 (10)	−0.0003 (10)	0.0018 (10)
N9	0.0525 (14)	0.0448 (14)	0.0472 (13)	0.0005 (11)	0.0199 (11)	0.0014 (10)
C10	0.077 (2)	0.0510 (18)	0.0426 (15)	0.0078 (16)	0.0224 (15)	−0.0060 (13)
C11	0.077 (2)	0.0523 (18)	0.0336 (14)	−0.0028 (16)	−0.0017 (13)	−0.0027 (13)
N12	0.0427 (12)	0.0343 (12)	0.0404 (11)	−0.0002 (9)	0.0018 (9)	−0.0001 (9)

### Geometric parameters (Å, º)

**Table:**

Cu1—C1	1.979 (3)	C7—N8	1.484 (5)
Cu1—C2	1.976 (3)	C7—H7A	0.9700
Cu1—C3	1.993 (3)	C7—H7B	0.9700
Cu1—C4	1.986 (3)	C6A—C7A	1.47 (3)
Cu2—N5	1.999 (2)	C6A—H6A1	0.9700
Cu2—N8	2.009 (2)	C6A—H6A2	0.9700
Cu2—N9	2.005 (2)	C7A—N8	1.41 (2)
Cu2—N12	2.004 (2)	C7A—H7A1	0.9700
C1—N1	1.129 (4)	C7A—H7A2	0.9700
C2—N2	1.139 (4)	N8—H8A	0.9000
C3—N3	1.123 (4)	N8—H8B	0.9000
C4—N4	1.138 (4)	N9—C10	1.467 (4)
Na—N1^i^	2.361 (3)	N9—H9A	0.9000
Na—N2	2.356 (3)	N9—H9B	0.9000
Na—N3^ii^	2.367 (3)	C10—C11	1.499 (5)
Na—N4^iii^	2.344 (3)	C10—H10A	0.9700
N5—C6A	1.464 (18)	C10—H10B	0.9700
N5—C6	1.480 (5)	C11—N12	1.470 (4)
N5—H5A	0.9000	C11—H11A	0.9700
N5—H5B	0.9000	C11—H11B	0.9700
C6—C7	1.497 (7)	N12—H12A	0.9000
C6—H6A	0.9700	N12—H12B	0.9000
C6—H6B	0.9700		
			
C1—Cu1—C2	114.43 (10)	H6A1—C6A—H6A2	108.3
C1—Cu1—C3	101.82 (11)	C6—C7—N8	108.3 (4)
C1—Cu1—C4	111.06 (11)	C6A—C7A—N8	107 (2)
C2—Cu1—C3	109.40 (10)	C6—C7—H7A	110.0
C2—Cu1—C4	105.04 (11)	C6—C7—H7B	110.0
C3—Cu1—C4	115.42 (12)	C6A—C7A—H7A1	110.3
N5—Cu2—N9	175.03 (10)	C6A—C7A—H7A2	110.3
N5—Cu2—N12	93.29 (9)	N8—C7—H7A	110.0
N9—Cu2—N12	84.65 (10)	N8—C7—H7B	110.0
N5—Cu2—N8	84.59 (10)	N8—C7A—H7A1	110.3
N9—Cu2—N8	97.23 (11)	N8—C7A—H7A2	110.3
N12—Cu2—N8	176.43 (10)	H7A—C7—H7B	108.4
N1^i^—Na—N2	107.11 (10)	H7A1—C7A—H7A2	108.6
N1^i^—Na—N3^ii^	100.77 (11)	C7—N8—Cu2	109.4 (2)
N1^i^—Na—N4^iii^	106.49 (12)	C7A—N8—Cu2	107.3 (10)
N2—Na—N3^ii^	109.50 (11)	C7—N8—H8A	109.8
N2—Na—N4^iii^	101.61 (11)	C7—N8—H8B	109.8
N3^ii^—Na—N4^iii^	129.84 (11)	C7A—N8—H8A	86.4
Cu1—C1—N1	172.9 (3)	C7A—N8—H8B	131.8
Cu1—C2—N2	173.5 (3)	Cu2—N8—H8A	109.8
Cu1—C3—N3	174.5 (3)	Cu2—N8—H8B	109.8
Cu1—C4—N4	173.8 (3)	H8A—N8—H8B	108.3
C1—N1—Na^iv^	137.0 (3)	Cu2—N9—C10	107.72 (18)
C2—N2—Na	147.6 (2)	Cu2—N9—H9A	110.2
C3—N3—Na^v^	113.3 (2)	Cu2—N9—H9B	110.2
C4—N4—Na^iii^	165.3 (3)	C10—N9—H9A	110.2
Cu2—N5—C6	108.9 (2)	C10—N9—H9B	110.2
Cu2—N5—C6A	105.6 (8)	H9A—N9—H9B	108.5
C6—N5—H5A	109.9	N9—C10—C11	107.6 (3)
C6—N5—H5B	109.9	N9—C10—H10A	110.2
C6A—N5—H5A	85.3	N9—C10—H10B	110.2
C6A—N5—H5B	134.0	C11—C10—H10A	110.2
Cu2—N5—H5A	109.9	C11—C10—H10B	110.2
Cu2—N5—H5B	109.9	H10A—C10—H10B	108.5
H5A—N5—H5B	108.3	C10—C11—N12	107.9 (2)
N5—C6—C7	107.9 (4)	C10—C11—H11A	110.1
N5—C6A—C7A	108.6 (19)	C10—C11—H11B	110.1
N5—C6—H6A	110.1	N12—C11—H11A	110.1
N5—C6—H6B	110.1	N12—C11—H11B	110.1
N5—C6A—H6A1	110.0	H11A—C11—H11B	108.4
N5—C6A—H6A2	110.0	C11—N12—Cu2	108.96 (18)
C7—C6—H6A	110.1	C11—N12—H12A	109.9
C7—C6—H6B	110.1	C11—N12—H12B	109.9
C7A—C6A—H6A1	110.0	Cu2—N12—H12A	109.9
C7A—C6A—H6A2	110.0	Cu2—N12—H12B	109.9
H6A—C6—H6B	108.4	H12A—N12—H12B	108.3
			
N5—C6—C7—N8	−50.3 (6)	N9—C10—C11—N12	53.0 (3)
N5—C6A—C7A—N8	58 (3)		

Symmetry codes: (i) *x*−1, *y*, *z*; (ii) −*x*+1, *y*−1/2, −*z*+1/2; (iii) −*x*+1, −*y*, −*z*+1; (iv) *x*+1, *y*, *z*; (v) −*x*+1, *y*+1/2, −*z*+1/2.
